# Osteo-regeneration personalized for children by rapid maxillary expansion: an imaging study based on synchrotron radiation microtomography

**DOI:** 10.1186/s12903-018-0590-7

**Published:** 2018-07-25

**Authors:** Alessandra Giuliani, Serena Mazzoni, Carlo Mangano, Piero Antonio Zecca, Alberto Caprioglio, Nicolò Vercellini, Mario Raspanti, Francesco Mangano, Adriano Piattelli, Giovanna Iezzi, Rosamaria Fastuca

**Affiliations:** 1 Sezione di Biochimica, Biologia e Fisica Applicata, Department of Clinical Sciences, Università Politecnica delle Marche, Via Brecce Bianche 1, 60131 Ancona, Italy; 2 Private Practice, Gravedona, CO Italy; 3 Department of Medicine and Surgery, University of Insubria, Via Guicciardini 9, Varese, Italy; 4 Department of Medical, Oral and Biotechnological Sciences, University of Chieti-Pescara, Via dei Vestini 31, 66100 Chieti Scalo, CH Italy; 5 Department of Biomedical Sciences, Dentistry and Morphological and Functional Imaging, University of Messina, Messina, Italy

**Keywords:** Rapid maxillary expansion, Medical imaging, Bone regeneration, Synchrotron radiation, Microtomography, Midpalatal suture

## Abstract

**Background:**

Personalized maxillary expansion procedure has been proposed to correct maxillary transversal deficiency; different protocols of stem cell activation have been suggested and rapid maxillary expansion (RME) is the most commonly used among clinicians. The present study aimed to quantify in three-dimensions (3D) the osteo-regeneration of the midpalatal suture in children submitted to RME.

**Methods:**

Three patients (mean age 8.3 ± 0.9 years) were enrolled in the study to preform biopsy of midpalatal suture. Two patients (subjects 1 and 2) were subjected to RME before biopsy. The third patient did not need maxillary expansion treatment and was enrolled as control (subject 3). Midpalatal suture samples were harvested 7 days after RME in subject 1, and 30 days after RME in subject 2. The samples were harvested with the clinical aim to remove bone for the supernumerary tooth extraction. When possible, maxillary suture and bone margins were both included in the sample. All the biopsies were evaluated by complementary imaging techniques, namely Synchrotron Radiation-based X-ray microtomography (microCT) and comparative light and electron microscopy.

**Results:**

In agreement with microscopy, it was detected by microCT a relevant amount of newly formed bone both 7 days and 30 days after RME, with bone growth and a progressive mineralization, even if still immature respect to the control, also 30 days after RME. Interestingly, the microCT showed that the new bone was strongly connected and cross-linked, without a preferential orientation perpendicular to the suture’s long axis (previously hypothesized by histology), but with well-organized and rather isotropic 3D trabeculae.

**Conclusions:**

The microCT imaging revealed, for the first time to the authors’ knowledge, the 3D bone regeneration in children submitted to RME.

**Electronic supplementary material:**

The online version of this article (10.1186/s12903-018-0590-7) contains supplementary material, which is available to authorized users.

## Background

Personalized maxillary expansion procedure was proposed to correct maxillary transversal deficiency [1, 2] by splitting the midpalatal suture stimulating cell growth towards osteo-regeneration [3]. Different protocol of stem cell activation were suggested and rapid maxillary expansion (RME) is the most spread among clinicians. RME was recently indicated as treatment not only to solve transversal maxillary deficiency but for a variety of clinical conditions [4] since sagittal problems and underdevelopment of the midface might be the consequences of untreated transversal deficiency [5–11]. Moreover, occlusal disharmony and functional problems involving breathing pattern changes might derive from maxillary arch deficiency [12–14]. RME was then underlined to have positive effects not only in increasing maxillary arch perimeter but also on general health of growing patients, then increasing the potential of its indications [4, 15, 16].

When RME is performed, dental and skeletal changes occur producing an increase in the upper arch dimension. The appliance produces midpalatal suture splitting and the defect created is usually filled with new bone [17]. Since the very beginning of its use, the skeletal effects of RME on mid palatal suture were investigated with the means of radiographic techniques in 2-dimensions [18] and 3-dimensions with cone beam computed tomography (CBCT) [19, 20] in order to better understand the processes behind the healing of the suture and then preventing relapse with adequate treatment and retention time. Significant density reduction right after the active phase of expansion with an increase in the sutural density after 6-months retention was showed by Lione [21]. Indeed, the limit of radiographic investigations was the lacking of comprehension of real cellular activity but only the presence/absence of mineralized tissue might be documented. For this reasons morphologic and histologic studies were performed mainly on animals. Several of them [22–25] showed how the healing process is the combination of multiple steps with new bone and connective tissue formation and remodeling. In particular remodeling process were reported to be continuous and 3 to 4 weeks were not enough to restore the initial inter-digitated form of the mid palatal suture [22]. The first investigations on human being performed by Melsen [26, 27] collected samples of growing subjects during RME at different stages of treatment and compared them to autoptic material subjected to no treatment.

Recently, some of the authors of the present study reported a case analysis at 7 and 30 days from RME [28]. The preliminary histological results showed bone growth in the gap already after 7 days, with the healing process still ongoing after 30 days from RME.

Even though some evidence was assessed on the topic no strong conclusions might be drawn according to the results of a recent systematic review [29].

This fact could easily be expected because standard imaging techniques, like radiography and histology, which are routinely implemented for bone analysis, cannot fully match statistical requests, although they provide useful complementary information.

In particular, while histology provides qualitative analysis of the newly formed bone after RME, 3D structural data and the relative quantitative analysis on regenerated bone are difficult to obtain by this technique. Indeed, although in principle the 3D morphology of the new bone could be extracted by the analysis of serial sections of the biopsy, this approach is not the optimum because of the histological decalcification that the sample undergoes before the analysis.

Furthermore, X-ray medical radiology presents several limitations, also in this case due to its 2D nature: radiographs just provide 2D images of a 3D object, not completely reconstructing the anatomy that is being assessed. Anatomical structures give superimposing signals, often with anatomical or background noises inducing difficulties in interpreting data. Usually, 2D radiographs show fewer details than those actually present, precluding also the analysis of the soft-tissue to hard-tissue relationships [30].

In this survey, the impact of the computed tomography (CT) technique has been revolutionary, enabling to study the bone with a contrast discrimination up to three orders of magnitude better than conventional radiography [31].

Absorption-based tomography, at high resolutions, i.e. microtomography (microCT), was demonstrated to give fundamental information on bone tissues microstructure, with images of the 3D spatial organization of the bone in different environmental [32–35] and genetic [36–38] conditions. Moreover, interesting microCT studies have been performed on different biomaterials, indicated as bone-substitute candidates, in dental [39, 40] and orthopedic [41, 42] districts, within an acellular strategy [43, 44] or combining the biomaterial with cells in vitro [45–48].

The availability of synchrotron radiation (SR) x-ray sources has further stimulated research based on the use of microCT. SR shows numerous advantages with respect to laboratory x-ray sources, including higher beam intensity, higher spatial coherence, and monochromaticity. In fact, the polychromatic source and cone-shaped beam geometry, like in CBCT, complicate assessment of bone mineral density. Depending the X-rays absorption on the amount of mineral in bone, a suitable calibration at SR facilities is able to correlate the reconstructed gray levels – in microCT images, obtained using a monochromatic X-ray beam, to the local bone mineral density [49].

The present study aimed to investigate, for the first time to the authors’ knowledge by SR-based microCT, the 3D changes in-vivo in midpalatal suture in humans, 7 and 30 days after RME.

This work exploits the monochromaticity property of SR, reducing the beam hardening effects, and simplifying the segmentation process of the images analysis.

We demonstrated that SR-based microCT, combined with a monochromatic X-ray beam, allows to study the early stages of bone regeneration in midpalatal suture, even on a very small cohort thanks to the 3D nature of the microCT analysis.

## Methods

### Subjects

Subjects presenting at the Division of Orthodontics (University of Insubria, Varese, Italy) and looking for orthodontic care were enrolled in the present study. The research protocol was reviewed and approved by the Ethical Committee of the AO Ospedale di Circolo e Fondazione Macchi (Varese, Italy), with Deliberative Act nr.826 of the 3rd of October, 2013. Moreover, the followed procedures adhered to the World Medical Organization Declaration of Helsinki. The parents of all the patients signed an informed consent for the enrollment of the children in the study and for the release of diagnostic documents for scientific purposes, before entering the treatment. All the patients had to comply with the following inclusion criteria to be enrolled in the study: 1) good general health as assessed with medical history and clinical judgement [50]; 2) patients who presented a supernumerary tooth located at the maxillary midline which had caused anomalies in the position of the upper incisors and for this reason need to be surgically removed. Indeed, the present sample was enrolled for the presence of a median maxillary supernumerary unerupted tooth (mesiodens) in mixed dentition, which had to be removed since causing eruption problems to the upper incisors in each single case.

**Fig. 1:**
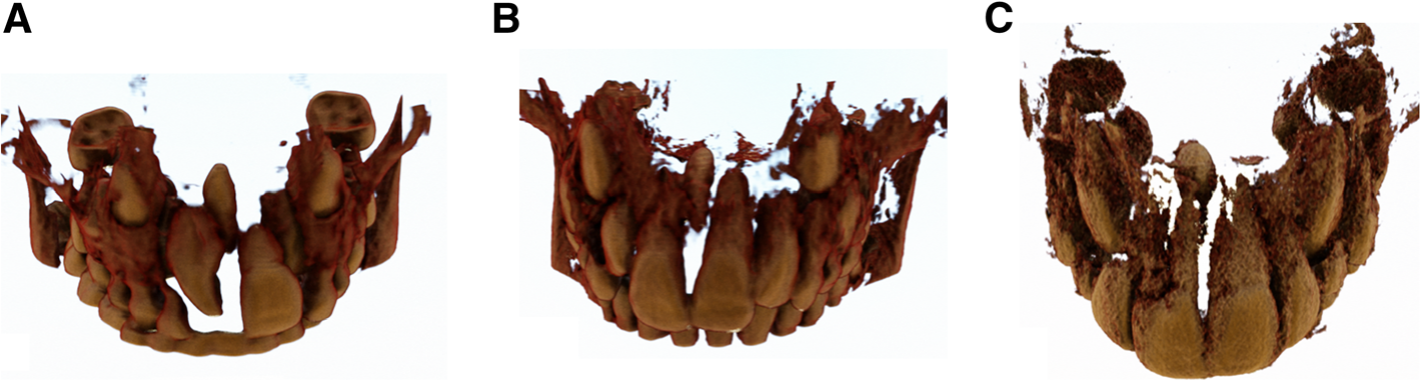
Volume rendering of the pretreatment cbct: (**a** and **b**) treated patients; (**c**) control patient

The surgical procedure of mesiodens extraction was made easier by the maxillary expansion, when needed, since the bone around the mesiodens was softer after the treatment. The bone or woven bone around the mesiodens was collected instead of the traditional demolition due to the bur in order to expose the mesiodens and perform the extraction and used as sample of the present study. Three patients (1 female and 2 males, mean age 8.3 ± 0.9 years) were enrolled in the study. Two patients (1 female, subject 1 and 1 male, subject 2) presented maxillary transverse deficiency that needed to be corrected with RME treatment before the supernumerary tooth extraction thus facilitating surgical procedure by reducing the amount of bone around the extraction site. The third patient did not need RME treatment but was enrolled as control (subject 3) since the supernumerary tooth on the maxillary midline was present. Each patient underwent CBCT recording (CS 9300, Carestream Dental, Atlanta, GA, USA) performed in seated position (120 kV, 3.8 mA, 30 s) [51] prior to the surgical treatment to accurately plan the surgery (Fig. 1).

Hyrax type expander (10-mm screw, A167–1439, Forestadent, Pforzheim, Germany) banded to the upper second deciduous molars as alternative to anchorage on permanent molars or miniscrews [52–54] was employed. The screw of the palatal expander was turned two times the day of its placement (0.45 mm initial transversal activation). Afterwards, parents of the patients were instructed to turn the screw once per each following day (0.225 mm activation per day). The maxillary expansion was performed until dental overcorrection. The expander was then kept on the teeth as a passive retainer and the patients underwent no further orthodontic treatment during retention.

### Biopsy procedure of the midpalatal suture

Midpalatal suture biopsies were collected during surgical removal of the supernumerary tooth in each patient. Contamination was avoided as much as possible by removing pathological tissue only after the biopsy of the midpalatal suture. After gathering of the mucous membrane of the hard palate, the biopsy was harvested by means of a cylindrical trephine bur with 7-mm on the midline along the midpalatal suture. Samples included both tissue sutures and one-side bone margin.

The treatment was performed only on subjects 1 and 2, while subject 3, not having received any treatment, was included as control. Each patient underwent a single biopsy collection, 7 days (subject 1) and 30 days (subject 2) after RME. The subject 3 (control) underwent surgery for mesiodens extraction and midpalatal suture biopsy, without any other treatment.

Then, the three biopsies were dehydrated in a glycolmethacrylate resin (Technovit 7200 VLC, Kulzer, Wertheim, Germany) to be investigated by microCT.

### Synchrotron radiation – based microtomography

The X-ray microCT scans were performed at the SYRMEP beamline of the ELETTRA synchrotron radiation facility (Trieste, Italy). The samples were investigated using isometric voxel with edge size of 4.2 μm; exposure time of 1600 ms/projection; and X-ray beam energy of 21 keV. The sample-detector distance of 50 mm enabled to work in absorption mode, where the resulting images were based solely on attenuation contrast.

The SYRMEP Tomo Project (STP) in-house software suite was used to reconstruct the tomographic slices, applying directly the standard filtered back-projection algorithm [55]. The STP is composed by a newly developed code and by external libraries [56].

The different grey values shown in the histogram of the reconstructed volumes are proportional to the linear attenuation coefficient μ of the different phases included in the sample, in turn proportional to their respective densities. The commercial software VG Studio MAX 1.2 (Volume Graphics, Heidelberg, Germany) was used to generate images for the visualization of the density distribution in 3D. Scatter HQ algorithm and an oversampling factor of 5.0 were considered the best settings to improve the x-ray contrast differences within samples. The volume of the bone was computationally obtained by multiplying the volume of a voxel (~ 74 μm^3^) by the number of voxels underlying the peak associated with it, after thresholding of the histograms by the Mixture Modeling Algorithm (MMA-NIH ImageJ Plugin). Indeed, thresholding was performed to automatically separate the newly formed bone phase from background and organic phase.

Structural analysis of the newly formed trabecular bone was performed in order to verify how the 3D morphology modifies from 7 to 30 days after RME. The following morphometric parameters were evaluated: Total Specific Volume (BV/TV – expressed as a percentage); Total Specific Surface (BS/BV – per millimeter); Mean Struts Thickness (BTh - expressed in micrometers); Mean Struts Number (BNr – per millimeter); Mean Struts Separation (BSp - expressed in micrometers); Anisotropy Degree (DA); Connectivity Density, i.e. number of trabeculae per unit volume (Conn.D. – expressed in pixel^− 3^).

The Degree of Anisotropy (DA) is a measure of how highly oriented the structures are within a certain volume. Indeed, trabecular bone structures could vary their orientation depending on time from RME. The DA index can vary between 0 (all observation confined to a single plane or axis) and 1 (perfect isotropy). DA of the retrieved samples, that is, the presence of preferential orientations, was analysed using the BoneJ Plugin [57] of ImageJ software (http://imagej.nih.gov/ij) [58], version 3.

For a faster visualization, 3D meshes were also obtained in standard Wavefront OBJ format with the commercial software *Mimics 17* (http://biomedical.materialise.com) and visualized with *Meshlab v1.3.3* (http:(//meshlab.sourceforge.net).

### Histological processing

After the microCT imaging, the sample blocks were prepared for the histological analysis. They were sectioned along the longitudinal axis, with a high precision diamond disk at about 150 μm and reduced to about 30 μm of thickness with the grinding machine Precise 1 Automated System (Assing, Rome, Italy). Three slices were prepared for each biopsy, that were stained with acid fuchsin and toluidine blue and imaged with a light microscope (Laborlux S, Leitz, Wetzlar, Germany) equipped with a high-resolution video camera (3CCD, JVC KY-F55B, JVC®, Yokohama, Japan) connected to a dedicated PC (Intel Pentium III 1200 MMX, Intel®, Santa Clara, CA, USA). The system was associated with a digitizing pad (Matrix Vision GmbH, Oppenweiler, Germany) and a software (Image-Pro Plus 4.5, Media Cybernetics Inc., Rockville, MD, USA) dedicated to histomorphometric analysis.

### Scanning electron microscopy

The Scanning Electron Microscopy (SEM) analysis of the specimens was carried out at the Laboratory of Human Morphology of the Insubria University. The blocks remaining after the preparation of the ground sections were mounted on appropriate stubs with conductive glue, carbon coated with an Emitech K550 sputter-coater (Quorum Emitech, Ashford, UK) fitted with an Emitech K250 flash evaporator (Quorum Emitech, Ashford, UK) and observed with a FEI XL-30 FEG high resolution Scanning Electron Microscope (FEI, Eindhoven, The Netherland) operating in Backscattered Electrons (BSE) imaging at an acceleration voltage of 20 kV. With this technique, the contrast formation depends on the local composition: in particular, the higher the atomic number the higher the resulting brightness. With an appropriate setting the mineralized regions stand out brightly against the soft matrix and the embedding resin. Pictures were directly obtained in digital format as 1424 × 968, 8bpp TIFF grayscale files.

### Data and statistical analysis

Morphometric data were statistically analysed with the support of the SigmaStat 3.5 software (Systat Software, San Jose, California). Statistical significance was assessed by two-tailed t test. *P*-values were considered significant when < 0.05.

## Results

### Synchrotron radiation - based microtomography

Osteo-regeneration of midpalatal suture sites, 7 and 30 days after RME, was studied by 3D microCT analysis.

**Fig. 2:**
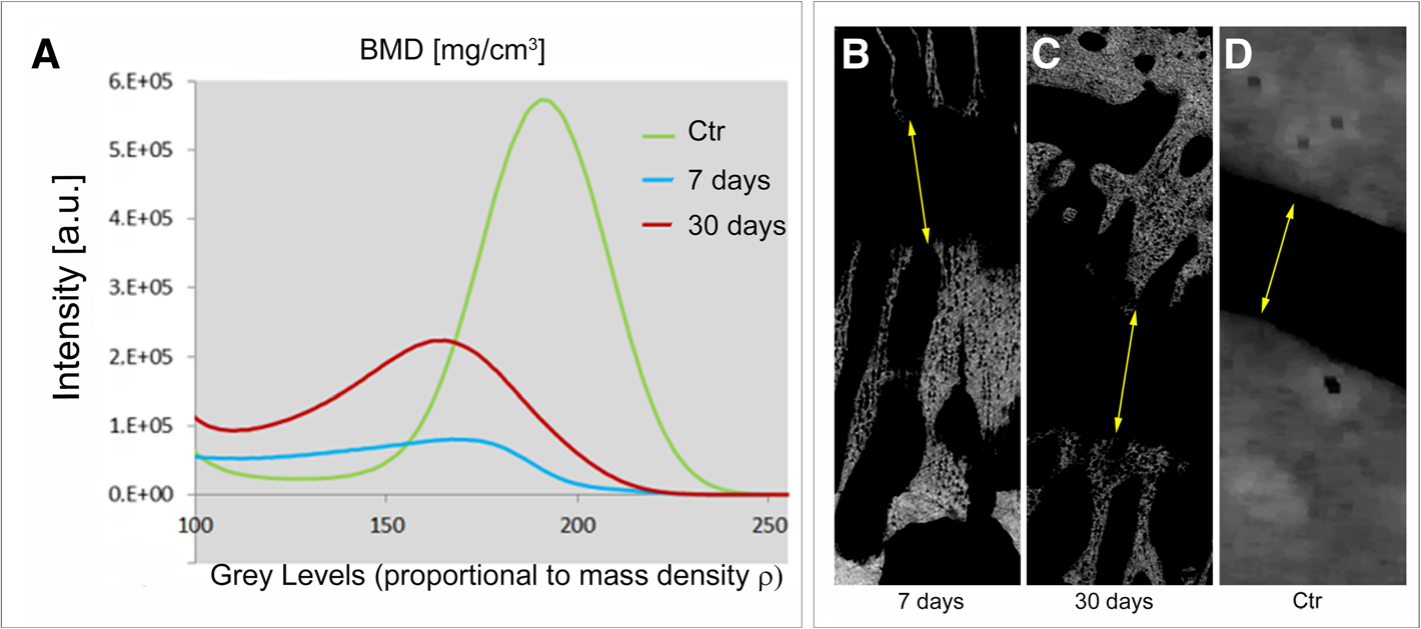
**a** Portion of the “intensity vs. gray levels” profile. The grey levels are proportional to the linear attenuation coefficient μ that, in turns, is nearly proportional to ρ, the bone mineral density (BMD). The integrated areas of the represented peaks correspond to the newly formed mineralized bone volume in RME-treated midpalatal sites and in the control. **b-d** Representative 2D sections of the treated palatal sites 7 days (**b**) and 30 days (**c**) after RME, and of the palatal control (**d**). The thickness of the suture channel was similar to that of the control suture (400–700 μm, yellow arrows), showing that the storiform way of remineralization was already started 7 days after RME

Figure 2 (panel a) reports the histogram referred to the bone mineralization degree (BMD- mg/cm^3^) study, respectively 7 and 30 days after the RME, comparing these profiles with the control midpalatal site. In these profiles, representing the “Intensity Counts vs. Grey Level”, the grey levels - here referred to an unsigned 8-bit scale - are proportional to the linear absorption coefficient μ, that in turn is nearly proportional to the BMD (i.e. the mass density) of the newly formed bone. Two different peaks were segmented, the first corresponding to air and soft tissues, and the other corresponding to the newly formed bone. The histogram area with the grey levels < 100, i.e. the area referred to air and soft tissues, was excluded by the present investigation. Independently from time of observation after RME, it was detected a relevant amount of bone in both the treated biopsies, as shown by the blue and red peaks, corresponding to the linear attenuation coefficient of the newly formed bone in biopsies retrieved 7 and 30 days after RME, respectively. While these peaks lie in a grey level range between 110 and 220, the control biopsy is in the range between 150 and 250, demonstrating that, 30 days after RME, the BMD in the treated sites is still sensibly lower than in the control site. Furthermore, the peaks referred to regenerated sites are broadened respect to the profile referred to the control, indicating a larger distribution of μ values strictly reasoned by the fact that the mineralization level is inhomogeneous during the midpalatal regeneration.

Representative 2D sections of these samples are shown in Fig. 2 (panels b, c, and d). Despite the similarity of the thickness of the suture channel already 7 days after RME compared to that of the control suture (400–700 μm, yellow arrows), the surrounding bone structure presented a storiform shape in the treated palates, against a bulky appearance in the control.

**Fig. 3:**
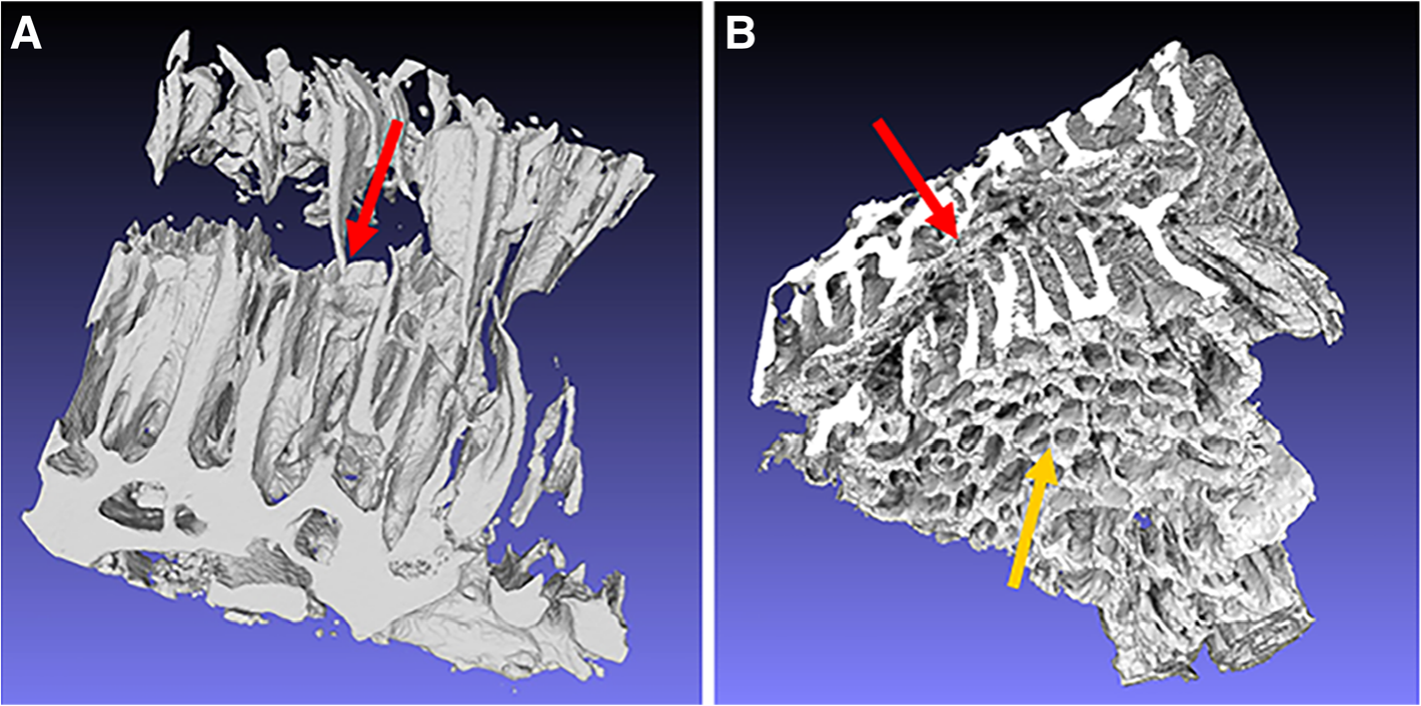
3D microCT rendering of the biopsies retrieved 7 days (**a**) and 30 days (**b**) after the RME. Both the specimens clearly showed the meshwork of the bone perforated by non-mineralized spaces. The direction indicated by the red arrows corresponded to the section plane of histological and SEM micrographs. The right image offers a better view of the canals (yellow arrows) that cross the whole thickness of the bone to reach the sutural channel


Moreover, as revealed by the 3D reconstructions (Fig. 3) and the Additional file 1: Video 1, the trabecular structures correspond to a sectioned grid of newly formed bone perforated by a regular lattice of spaces, structures that are supposed to maximize the contact of the vascular net with the growing calcified tissue.

**Additional file 1: Video 1.** Animation referred to the microCT 3D reconstruction of the biopsy got 30 days after RME. The movie clearly shows a strongly connected and cross-linked structure, similar to the morphology of a bone scaffold. The trabecular structures correspond to a sectioned grid of newly formed bone perforated by a regular lattice of spaces, structures that are supposed to maximize the contact of the vascular net with the growing calcified tissue. (AVI 59591 kb)


In order to estimate the evolution of these structures, a morphometric analysis of the overall 3D mineralized tissues was performed. The results are shown in Table 1.

**Table 1:** 3D morphometric analysis of the constructs retrieved 7 and 30 days after rapid maxillary expansion (RME). The characterization of the 3D mineralized microarchitecture of the newly formed bone showed that the struts number (BNr) significantly increase from 7 to 30 days after RME. Coherently, the spacing (BSp) significantly decrease and the Conn. D significantly increase from 7 to 30 days after RME

Morphometric Parameters	7 days	30 days	Significance Level (*P* value)
Total Specific Volume - BV/TV [%]	22.7 ± 7.3	29.5 ± 2.6	No, *P* > 0.05
Total Specific Surface - BS/BV [mm^− 1^]	47 ± 14	49 ± 7	No, *P* > 0.05
Mean Struts Thickness – BTh [μm]	45 ± 11	42 ± 6	No, *P* > 0.05
Mean Struts Number – BNr [mm^− 1^]	5.3 ± 0.6	7.3 ± 0.6	Yes, *P* = 0.013
Mean Struts Spacing – BSp [μm]	157 ± 30	99 ± 5	Yes, *P* = 0.028
Anisotropy Degree - DA	0.782 ± 0.097	0.758 ± 0.047	No, *P* > 0.05
Connectivity Density - Conn.D. (× 10^− 5^) [μm^-3^]	3.610 ± 1.651	7.618 ± 0.156	Yes, *P* = 0.014

This characterization showed that, even if not significant differences (*p* > 0.05) between specific volumes (BV/TV), specific surface (BS/BV) and mean trabecular thicknesses (BTh) were detected at the two time-points, the mean struts number (BNr) significantly increased from 7 days to 30 days after RME (*p* = 0.013). Coherently, the spacing (BSp) significantly decreased (*p* = 0.028).

The anisotropy analysis showed that, with respect to this parameter, despite the significant increasing of the struts number, the structure preserved its orientation from 7 days to 30 days from the treatment starting, suggesting a natural evolution of a regeneration process already started after the first week from RME. Furthermore, an average DA value of 0.7–0.8 indicated that the structure was highly isotropic in 3D.

As expected by the increased number of struts, also the Conn.D parameter significantly increased from 7 to 30 days after RME (*p* = 0.014), demonstrating that the structure became more and more bulky, with an expected trend in time towards the control morphology.

**Fig. 4:**
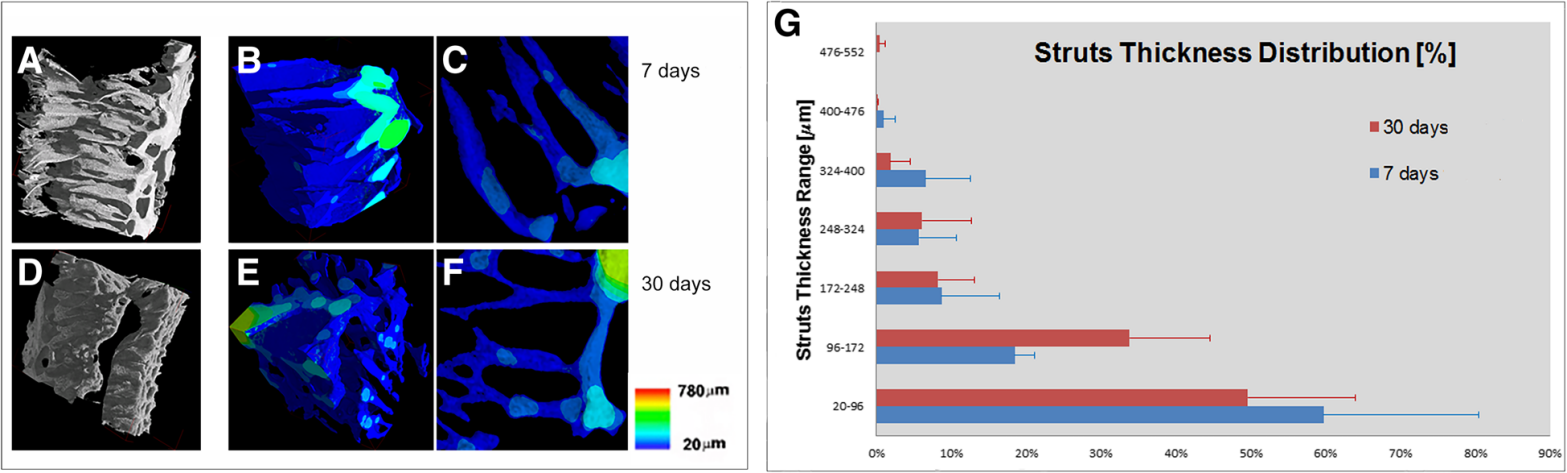
**a-c** Biopsy retrieved 7 days after RME: (**a**) 3D microCT reconstruction; (**b**) Study in 3D of the thickness distribution basing on a color map; (**c**) 2D sampling color mapped slice. **d-f** Biopsy retrieved 30 days after RME: (**d**) 3D microCT reconstruction; (**e**) Study in 3D of the thickness distribution basing on a color map; (**f**) 2D sampling color mapped slice. Thickness scale for the color map at the bottom-center position. **g** Histogram of the distribution of the newly formed bone thickness in both the RME-treated midpalatal biopsies. These data demonstrate that there was a slight (not significant; *p* > 0.05) increase in thickness of the struts from 7 days to 30 days after RME

To better visualize and compare the newly formed bone at 7 and 30 days after RME, the 3D color maps of the bone thickness distribution were also reconstructed, as shown in Fig. 4 (panels a-f).

The whole biopsies of the samples retrieved respectively 7 and 30 days after the RME treatment were shown in Fig. 3a and d. The same samples were visualized with different orientations respectively in Figs. 4b and 3e, better showing the 3D distribution of trabecular size, in agreement with the color bar in the bottom-center position of Fig. 4. The same information was better displayed in selected 2D slices, 7 (Fig. 4c) and 30 (Fig. 4f) days after the RME. An overall significant increase of the number of trabeculae and a slight increase of the trabecular thickness were observed from 7 to 30 days after RME. Indeed, the color maps demonstrated that there was a slight increase in thickness of the struts (as well as for the BNr) from 7 to 30 days after RME. To confirm this evidence, the “bone thickness distribution vs. the bone volume normalized to the total sample volume” was also assessed. The graph of the bone thickness distribution in both the investigated samples was reported in Fig. 4 panel g. It was shown here that, even if the average bone thickness was calculated to be similar, 7 days after RME there was a 10% of struts in the range between 20 and 96 μm more than 30 days after RME and, in the range between 96 and 172 μm, it was the opposite.

### Comparative microscopy results

#### Light microscopy

**Fig. 5:**
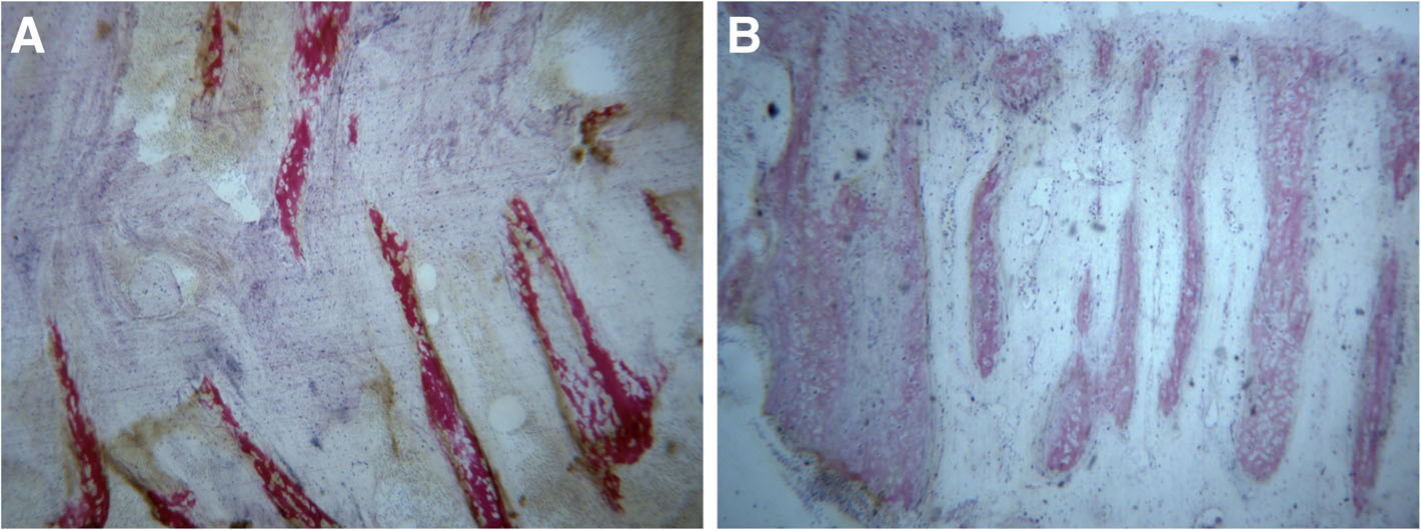
Light microscopy. **a** 7 days after rapid maxillary expansion: trabecular new bone with storiform appearance was observed. **b** 30 days after rapid maxillary expansion: the newly-formed bone trabeculae were oriented perpendicularly to the long axis of the suture. Toluidine blue and acid fuchsin were used. Original magnification 40×

Trabeculae apparently having storiform features and connective tissue were observed, 7 days after RME, inside the suture (Fig. 5a). They were composed by newly formed bone, with wide osteocyte lacunae. Small bone fibers were observed close to the blood vessels.

Thirty days after RME, more trabeculae were observed: they are closer than after 7 days from RME and, while in several fields they appeared aligned parallel to each other with a perpendicular orientation to the long axis of the suture, in other few fields they merged into one another (Fig. 5b). However, the rich osteoblastic activity and the detection of osteoid matrix undergoing mineralization in many areas suggested that the osteo-regeneration process was still not ended after 30 days from RME.

#### Scanning electron microscopy

SEM analysis allowed to achieve high-resolution 2D imaging of the planed face of the specimens, with a mechanism of contrast formation reminiscent of the microCT slices. By analogy with the microCT, with SEM operating in backscattered electron mode, the mineralized portion emerged clearly against the dark backdrop of soft tissue and resin.

**Fig. 6:**
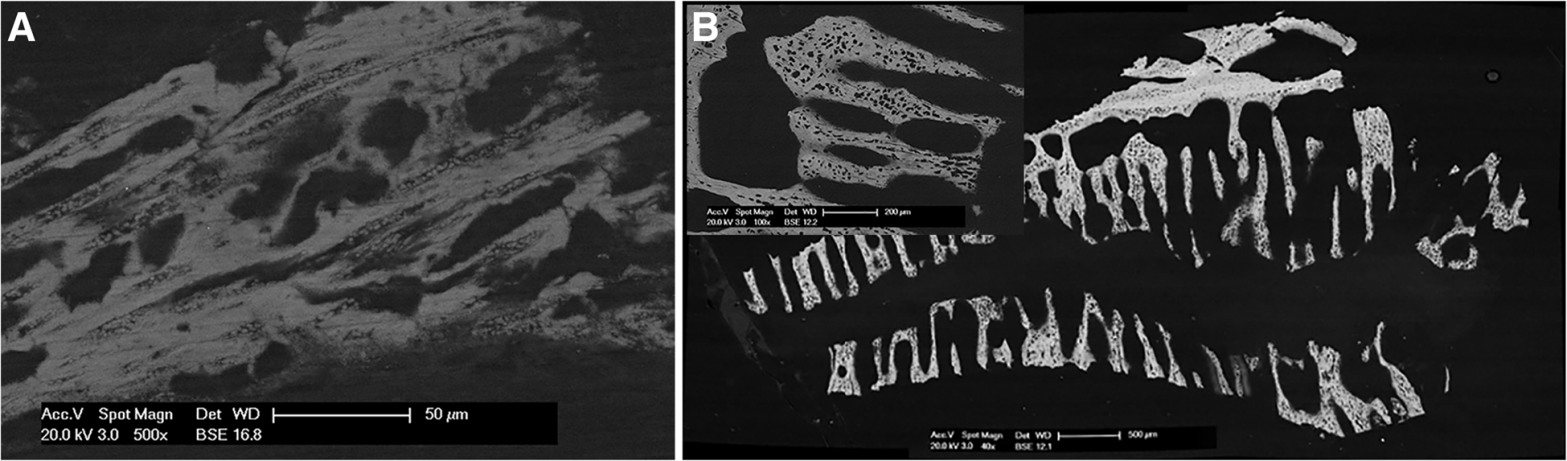
**a** Biopsy at 7 days from RME: detail of the tissue at very high magnification. Irregular osteocyte lacunae were interspersed with dark streaks; the tiny dust-like specks were distinct simultaneous loci of mineralization. Bar = 50 μm. **b** Biopsy at 30 days from RME: mosaic of five distinct SEM micrographs of an histological section. The suture, running left to right, was flanked on both sides by elongated streaks, perpendicular to the same suture, separated by empty spaces. Bar = 500 μm. Top-left inset: detail of the mineralized tissue at higher magnification, with irregular osteocyte lacunae. Bar = 200 μm

The SEM analysis of the biopsy 7 days after RME confirmed the results obtained by microCT and histology. Indeed, the bone matrix was observed to be traversed by dark longitudinal streaks, corresponding to zones of incomplete mineralization; the very high magnifications revealed simultaneous multiple loci of mineralization (Fig. 6a), consistent with a fast neoformation of bone towards the suture channel.

Low magnification images, like Fig. 6 - panel b showing the biopsy at 30 days from RME, were consistent with conventional histology and confirm the presence of elongated bone structures, dendrites, apparently perpendicular to suture axis, pointing towards the sutural space. Indeed, the SEM image in Fig. 6 (panel b), perfectly match the microCT morphologic information shown in Fig. 1c.

At higher magnification (top-left inset of Fig. 6b), in agreement with histologic findings, the calcified tissue exhibited large, irregular osteocyte lacunae, gathered in uneven clusters and suggestive of a fast, storiform growth.

## Discussion

Rapid midpalatal expansion effects on suture changes were of great clinical interest in the last years, with studies mainly focused on identifying and qualifying the immediate and long-term effects of this treatment in growing teenage or young adults by conventional imaging methods [29]. The specific aim of this case report was to study, for the first time to the authors’ knowledge, the short-term 3D quantitative changes after RME by Synchrotron radiation-based microCT.

A similar investigation, enrolling the same subjects, was reported in a previous case report [28]. However, the limit of this study was linked to the high morphometric variability of histological data.

As documented in literature [59], it is often suggested to couple 2D conventional microscopy with advanced 3D quantitative analysis. Indeed, with the use of microCT, it is reasonable to get significant morphometric results on a statistical sample sometimes narrower than the number of patients involved in the histologic study [60, 61], in these cases making no longer necessary the calculation of the statistical power.

In our study, microCT allowed to achieve significant quantitative results in spite of including a single subject for comparisons at 7 days, 30 days after RME, and a control. Indeed, the previous case report [28] on the same subjects was only descriptive and exclusively based on 2D data.

In agreement with histological findings, this microCT study detected a relevant amount of newly formed bone both 7 and 30 days after RME. Furthermore, as previously reported [28], it was observed a progressive mineralization with the peculiar in-plane fishbone appearance of the trabecular bone. As reported in literature [22, 28], the suture mineralization and morphology were confirmed in 3D to be still immature respect to the control, also 30 days after RME.

However, the microCT analysis did not confirm in 3D another finding observed in 2D by light and electron microscopy, i.e. that the newly formed bone trabeculae were oriented perpendicularly to the long axis of the suture and run parallel to each other [28]. Several microCT data contributed to denying in 3D this observation: the calculated value of DA, both 7 and 30 days after the RME, suggests a rather isotropic and poorly oriented structure; the combined significant increase in the number of trabeculae and their connectivity is not compatible with a structure consisting of parallel trabeculae. Moreover, the animation referred to the biopsy got 30 days after RME (delivered as Additional file 1: Video 1), clearly shows a strongly connected and cross-linked structure, similar to the morphology of a bone scaffold, that is expected to become more and more bulky, mimicking the control morphology.

## Conclusions

The microCT imaging revealed, for the first time to the authors’ knowledge, the following bone regeneration in children submitted to RME: few bone dendrites poorly connected after 7 days from the treatment, more dendrites and more connected after 30 days. Histologic and SEM 2D images showed portions of these dendrites, mainly oriented towards the suture channel, but the 3D microCT observations revealed also the interdendritic connections that, in turn, increased the overall isotropy of the structure, with possible beneficial implications in terms of biomechanical stability.

A drawback of the present study is to have stopped the experimental observations at 30 days from RME, when the microCT and the comparative techniques converge in asserting that the healing process has not yet ended at that time-point.

In synthesis, the morphometric data, as extracted by microCT analysis and 2D microscopy, converge to confirm the progressive healing process, activated by the endogenous stem cells, and the mineralization of trabecular bone structure. These microCT-imaging findings indicated that the new trabeculae might not be oriented perpendicularly to the long axis of the suture, as deduced by 2D microscopy in previous studies.

## Abbreviations

(2D): Two-dimensions
(3D): Three-dimensions
(CBCT): Cone beam computed tomograph
(microCT): Microtomography
(RME): Rapid maxillary expansion
(SR): Synchrotron

## References

[1] Angell EC (1860). Treatment of irregularities of the permanent or adult teeth. Dent Cosmos..

[2] Haas AJ (1965). The treatment of maxillary deficiency by opening the midpalatal suture. Angle Orthod.

[3] Pullen HA (1912). Expansion of the dental arch and opening the maxillary suture in relation to the development of the internal and external face. Dent Cosmos.

[4] McNamara JA, Lione R, Franchi L, Angelieri F, Cevidanes LH, Darendeliler MA (2015). The role of rapid maxillary expansion in the promotion of oral and general health. Prog Orthod.

[5] Eichenberger M, Baumgartner S (2014). The impact of rapid palatal expansion on children’s general health: a literature review. Eur J Paediatr Dent.

[6] Di Blasio A, Mandelli G, Generali I, Gandolfini M (2009). Facial aesthetics and childhood. Eur J Paediatr Dent.

[7] Di Blasio C, Di Blasio A, Pedrazzi G, Anghinoni M, Sesenna E (2015). How does the mandible grow after early high condylectomy?. J Craniofac Surg.

[8] Di Blasio A, Cassi D, Di Blasio C, Gandolfini M (2013). Temporomandibular joint dysfunction in Moebius syndrome. Eur J Paediatr Dent.

[9] Zecca PA, Fastuca R, Beretta M, Caprioglio A, Macchi A. Correlation assessment between three-dimensional facial soft tissue scan and lateral cephalometric radiography in orthodontic diagnosis. Int J Dent. 2016:1473918. 10.1155/2016/1473918 10.1155/2016/1473918 PMC4903122 27313615

[10] Bianchi B, Ferri A, Brevi B, Di Blasio A, Copelli C, Di Blasio C (2013). Orthognathic surgery for the complete rehabilitation of Moebius patients: principles, timing and our experience. J Craniomaxillofac Surg.

[11] Anghinoni ML, Magri AS, Di Blasio A, Toma L, Sesenna E (2009). Midline mandibular osteotomy in an asymmetric patient. Angle Orthod..

[12] Fastuca R, Perinetti G, Zecca PA, Nucera R, Caprioglio A (2015). Airway compartments volume and oxygen saturation changes after rapid maxillary expansion: a longitudinal correlation study. Angle Orthod..

[13] Fastuca R, Meneghel M, Zecca PA, Mangano F, Antonello M, Nucera R (2015). Multimodal airway evaluation in growing patients after rapid maxillary expansion. Eur J Paediatr Dent.

[14] Caprioglio A, Meneghel M, Fastuca R, Zecca PA, Nucera R, Nosetti L (2014). Rapid maxillary expansion in growing patients: correspondence between 3-dimensional airway changes and polysomnography. Int J Pediatr Otorhinolaryngol.

[15] Fastuca R, Zecca PA, Caprioglio A (2014). Role of mandibular displacement and airway size in improving breathing after rapid maxillary expansion. Prog Orthod.

[16] Caprioglio A, Bergamini C, Franchi L, Vercellini N, Zecca PA, Nucera R (2017). Prediction of class II improvement after rapid maxillary expansion in early mixed dentition. Prog Orthod.

[17] Dewey M (1914). Bone development as a result of mechanical force: report on further treatment in attempting the opening of the Intermaxillary suture in animals. Items Interest.

[18] da Silva Filho OG, Montes LA, Torelly LF (1995). Rapid maxillary expansion in the deciduous and mixed dentition evaluated through posteroanterior cephalometric analysis. Am J Orthod Dentofac Orthop.

[19] Franchi L, Baccetti T, Lione R, Fanucci E, Cozza P (2010). Modifications of midpalatal sutural density induced by rapid maxillary expansion: a low-dose computed-tomography evaluation. Am J Orthod Dentofac Orthop.

[20] Acar YB, Motro M, Everdi N (2015). Hounsfield units: a new indicator showing maxillary resistance in rapid maxillary expansion cases?. Angle Orthod..

[21] Lione R, Franchi L, Fanucci E, Laganà G, Cozza P (2013). Three-dimensional densitometric analysis of maxillary sutural changes induced by rapid maxillary expansion. Dentomaxillofac Radiol.

[22] Storey E (1955). Bone changes associated with tooth movement: a histological study of the effect of force in the rabbit, Guinea pig and rat. Aust Dent J.

[23] Cleall JF, Bayne DI, Posen JM, Subtenly JD (1965). Expansion of the Midpalatal suture in the monkey. Angle Orthod.

[24] Murray JM, Cleall JF (1971). Early tissue response to rapid maxillary expansion in the midpalatal suture of the rhesus moneky. J Dent Res.

[25] Ohshima O (1972). Effect of lateral expansion force on the maxillary structure in Cynomolgus monkey. J Osaka Dent Univ.

[26] Melsen B. A histological study of the influence of sutural morphology and skeletal maturation on rapid palatal expansion in children. Trans Eur Orthod Sot. 1972:499–507. 4596566

[27] Melsen B (1975). Palatal growth studied on human autopsy material. Am J Orthod.

[28] Caprioglio A, Fastuca R, Zecca PA, Beretta M, Mangano C, Piattelli A (2017). Cellular Midpalatal Suture Changes after Rapid Maxillary Expansion in Growing Subjects: A Case Report. Int J Mol Sci.

[29] Liu S, Xu T, Zou W (2015). Effects of rapid maxillary expansion on the midpalatal suture: a systematic review. Eur J Orthod.

[30] Shah N, Bansal N, Logani A (2014). Recent advances in imaging technologies in dentistry. World J Radiol.

[31] Claesson T (2001). A medical imaging demonstrator of computed tomography and bone mineral densitometry.

[32] Tavella S, Ruggiu A, Giuliani A, Brun F, Canciani B, Manescu A (2012). Bone turnover in wild type and pleiotrophin-transgenic mice housed for three months in the international Space Station (ISS). PLoS One.

[33] Canciani B, Ruggiu A, Giuliani A, Panetta D, Marozzi K, Tripodi M (2015). Effects of long time exposure to simulated micro- and hypergravity on skeletal architecture. J Mech Behav Biomed Mater.

[34] Shiba D, Mizuno H, Yumoto A, Shimomura M, Kobayashi H, Morita H (2017). Development of new experimental platform 'MARS'-multiple artificial-gravity research system-to elucidate the impacts of micro/partial gravity on mice. Sci Rep.

[35] Gerbaix M, Gnyubkin V, Farlay D, Olivier C, Ammann P, Courbon G (2017). One-month spaceflight compromises the bone microstructure, tissue-level mechanical properties, osteocyte survival and lacunae volume in mature mice skeletons. Sci Rep.

[36] Costa D, Lazzarini E, Canciani B, Giuliani A, Spanò R, Marozzi K (2014). Altered bone development and turnover in transgenic mice over-expressing lipocalin-2 in bone. J Cell Physiol.

[37] Hoshino M, Uesugi K, Yagi N (2012). Phase-contrast X-ray microtomography of mouse fetus. Biology Open.

[38] Jiang Y, Zhao J, Liao EY, Dai RC, Wu XP, Genant HK (2005). Application of micro-CT assessment of 3-D bone microstructure in preclinical and clinical studies. J Bone Miner Metab.

[39] Iezzi G, Piattelli A, Giuliani A, Mangano C, Barone A, Manzon L (2017). Molecular, cellular and pharmaceutical aspects of bone grafting materials and membranes during maxillary sinus-lift procedures. Part 2: detailed characteristics of the materials. Curr Pharm Biotechnol.

[40] Rominu M, Manescu A, Sinescu C, Negrutiu ML, Topala F, Rominu RO, et al. Zirconia enriched dental adhesive: A solution for OCT contrast enhancement. Demonstrative study by synchrotron radiation microtomography. Dent Mater. 2014;30(4):417–23. 10.1016/j.dental.2014.01.004 24530139

[41] Cancedda R, Cedola A, Giuliani A, Komlev V, Lagomarsino S, Mastrogiacomo M (2007). Bulk and interface investigations of scaffolds and tissue-engineered bones by X-ray microtomography and X-ray microdiffraction. Biomaterials.

[42] Atwood RC, Jones JR, Lee PD, Hench LL (2004). Analysis of pore interconnectivity in bioactive glass foams using X-ray microtomography. Scripta Mater.

[43] Giuliani A, Manescu A, Larsson E, Tromba G, Luongo G, Piattelli A (2014). In vivo regenerative properties of coralline-derived (biocoral) scaffold grafts in human maxillary defects: demonstrative and comparative study with Beta-Tricalcium phosphate and biphasic calcium phosphate by synchrotron radiation X-ray microtomography. Clin Implant Dent Relat Res.

[44] Giuliani A, Manescu A, Mohammadi S, Mazzoni S, Piattelli A, Mangano F (2016). Quantitative kinetics evaluation of blocks versus granules of biphasic calcium phosphate scaffolds (HA/β-TCP 30/70) by synchrotron radiation X-ray microtomography: a human study. Implant Dent.

[45] Komlev VS, Peyrin F, Mastrogiacomo M, Cedola A, Papadimitropoulos A, Rustichelli F (2006). Kinetics of in vivo bone deposition by bone marrow stromal cells into porous calcium phosphate scaffolds: an X-ray computed microtomography study. Tissue Eng.

[46] Manescu A, Giuliani A, Mazzoni S, Mohammadi S, Tromba G, Diomede F (2016). Osteogenic potential of dual-blocks cultured with periodontal ligament stem cells: in-vitro and synchrotron microtomography study. J Periodontal Res.

[47] Mazzoni S, Mohammadi S, Tromba G, Diomede F, Piattelli A, Trubiani O (2017). Role of cortico-cancellous heterologous bone in human periodontal ligament stem cell xeno-free culture studied by synchrotron radiation phase-contrast microtomography. Int J Mol Sci.

[48] Komlev VS, Mastrogiacomo M, Pereira RC, Peyrin F, Rustichelli F, Cancedda R (2010). Biodegradation of porous calcium phosphate scaffolds in an ectopic bone formation model studied by X-ray computed microtomograph. Eur Cell Mater.

[49] Kazakia GJ, Burghardt AJ, Cheung S, Majumdar S (2008). Assessment of bone tissue mineralization by conventional x-ray microcomputed tomography: comparison with synchrotron radiation microcomputed tomography and ash measurements. Med Phys.

[50] Biondi K, Lorusso P, Fastuca R, Mangano A, Zecca PA, Bosco M (2016). Evaluation of masseter muscle in different vertical skeletal patterns in growing patients. Eur J Paediatr Dent.

[51] Cassi D, De Biase C, Tonni I, Gandolfini M, Di Blasio A, Piancino MG (2016). Natural position of the head: review of two-dimensional and three-dimensional methods of recording. Br J Oral Maxillofac Surg.

[52] Fontana M, Cozzani M, Caprioglio A (2012). Non-compliance maxillary molar distalizing appliances: an overview of the last decade. Prog Orthod.

[53] Caprioglio A, Fontana M, Longoni E, Cozzani M (2013). Long-term evaluation of the molar movements following pendulum and fixed appliances. Angle Orthod..

[54] Giuliano Maino B, Pagin P, Di Blasio A (2012). Success of miniscrews used as anchorage for orthodontic treatment: analysis of different factors. Prog Orthod.

[55] Kak AC, Slaney M (2001). Principles of computerized tomographic imaging. Society of Industrial and Applied Mathematics.

[56] Brun F, Pacilè S, Accardo A (2015). Enhanced and flexible software tools for X-ray computed tomography at the Italian synchrotron radiation facility Elettra. Fundamenta Informaticae.

[57] Doube M, Klosowski MM, Arganda-Carreras I, Cordelières FP, Dougherty RP, Jackson JS (2010). BoneJ: free and extensible bone image analysis in ImageJ. Bone.

[58] Schneider CA, Rasband WS, Eliceiri KW (2012). NIH image to ImageJ: 25 years of image analysis. Nat Methods.

[59] Giuliani A. Analysis of bone response to dental bone grafts by advanced physical techniques. In: Piattelli A, editor. Bone response to dental implant materials: Elsevier Ltd; 2016. p. 229–46. 10.1016/B978-0-08-100287-2.00012-4.

[60] Suresh KP, Chandrashekara S (2012). Sample size estimation and power analysis for clinical research studies. J Hum Reprod Sci.

[61] Giuliani A, Iezzi G, Mazzoni S, Piattelli A, Perrotti V, Barone A (2018). Regenerative properties of collagenated porcine bone grafts in human maxilla: demonstrative study of the kinetics by synchrotron radiation microtomography and light microscopy. Clin Oral Investig.

